# Whole genome sequencing to inform the epidemiology of *Plasmodium falciparum* malaria in the elimination setting of Malaysia

**DOI:** 10.1186/s12864-026-12653-7

**Published:** 2026-02-20

**Authors:** Mark K. I. Tan, Nina Billows, Paul C. S. Divis, Cyrus Daneshvar, Jonathan Edgeworth, Janet Cox Singh, Susana Campino, Taane G. Clark

**Affiliations:** 1 Department of Infection Biology, Faculty of Infectious and Tropical Diseases, London School of Hygiene and Tropical Medicine (LSHTM), London, WC1E 7HT UK; 2 Department of Infectious Diseases, Faculty of Life Sciences and Medicine, King’s College London, London, UK; 3 Department of Infection, Centre for Clinical Infection and Diagnostics Research, Guy’s & St. Thomas’ NHS, London, SE1 7EH UK; 4 Faculty of Medicine and Health Sciences, Malaria Research Centre, Universiti Malaysia Sarawak, Kota Samarahan, Sarawak Malaysia; 5 University Hospitals Plymouth NHS Trust, Plymouth, UK; 6 School of Medicine, University of St Andrews, North Haugh, St Andrews, KY16 9TF UK; 7 Faculty of Epidemiology and Population Health, London School of Hygiene and Tropical Medicine, LSHTM, London, WC1E 7HT UK

**Keywords:** *Plasmodium falciparum*, Malaria, Nanopore sequencing technology, Imported diseases, Malaysia, Molecular epidemiology, Population surveillance

## Abstract

**Background:**

Imported malaria cases, driven by human migration and travel, pose a significant challenge to malaria elimination efforts. Genomic approaches have become essential for distinguishing between local transmission and imported infections. The state of Sarawak, Malaysia, provides a pertinent example of a malaria-eliminating setting under pressure from *Plasmodium* parasite importation.

**Results:**

In this study, we analysed 21 *Plasmodium falciparum* isolates obtained from archived whole blood samples collected between 2008 and 2010 and compared them to 9,518 publicly available isolates from Central Africa (518), East Africa (849), Horn of Africa (25), Oceania (349), South America (75), South Asia (404) Southeast Asia (5,182) and West Africa (2,116). By applying nanopore sequencing and population genomic analyses, we found that most of the cases (*n* = 13/15) likely originated from endemic regions outside Malaysia, supported by patient travel histories and high multiplicity of infection levels. These findings and drug resistance profiles are consistent with the historical epidemiology of the suspected source regions. Notably, two cases showed genomic evidence of origins inconsistent with the patients’ reported travel histories, underscoring the limitations of traditional epidemiological methods. Identity-by-descent analysis revealed clustering in only two cases, indicating that the majority of infections were likely isolated introductions rather than evidence of sustained local transmission.

**Conclusion:**

Overall, our results highlight the power of malaria genomics in discerning imported from locally acquired cases and emphasise its critical role in maintaining malaria elimination, particularly in regions situated along major migration and labour exchange corridors.

**Supplementary Information:**

The online version contains supplementary material available at 10.1186/s12864-026-12653-7.

## Introduction

Malaria, caused by *Plasmodium* parasites and transmitted by *Anopheles* mosquitoes, has a high global burden, with over 282 million estimated cases and 610,000 associated deaths in 2024 alone [1]. Despite these challenges, progress towards elimination has been significant. A total of 47 countries have been certified malaria-free by the World Health Organization (WHO) - a designation granted to countries that demonstrate interruption of local malaria transmission for at least three consecutive years and maintain a robust surveillance system to prevent re-establishment [1]. Malaria control measures, such as the use of insecticides, insecticide-treated nets, and antimalarial medications like artemisinin-based combination therapies, have significantly reduced the disease burden. However, challenges, including zoonotic malaria, drug resistance, and climate change, are hindering further progress toward elimination [1].

Malaria elimination is focused on controlling the local transmission of human-host adapted *Plasmodium* species. Paths to malaria elimination can be undermined by the importation of cases from endemic regions into increasingly immune-naïve populations, formerly endemic for malaria. Importation can be driven by migration, travel, and re-introduction through asymptomatic parasite reservoirs [2]. A good example of malaria importation is southeastern Iran, where indigenous transmission was limited, but has experienced malaria resurgence linked to importation from neighbouring countries [3]. In Malaysia, where *Plasmodium falciparum* transmission has been largely interrupted through sustained control efforts, the risk of re-establishment persists due to the influx of migrant workers from malaria-endemic countries [4]. Similarly, countries such as Cambodia, Nepal, and Bhutan, despite substantial declines in malaria incidence, continue to face challenges from the sporadic detection of indigenous *P. falciparum* cases in areas previously considered malaria-free [2].

National malaria control programmes (NMCPs) are using genomics tools to investigate outbreaks [5]. For example, imported cases of malaria into non-endemic countries, including the UK, are being investigated by applying genomics-based tools to identify *Plasmodium* species, drug resistance markers and the likely source of infections [6]. Whole-genome (WGS) or targeted amplicon sequencing data can provide insights into drug resistance, multiplicity of infection linked to transmission intensity, and the geographical source of infections that can identify importation events, all supported by informatics tools [6]. Population genomic analysis elucidates population structure and ancestry, transmission events and parasite relatedness through identity-by-descent (IBD), and intervention effectiveness [7]. Decreasing sequencing costs, including developments in Oxford Nanopore Technologies (ONT) portable sequencing platforms, as well as selective whole genome amplification (SWGA) of DNA for low parasitaemia infections [8], are leading to NMCPs investing in sequencing technologies to improve genomic resolution to characterise spatial and temporal trends in drug resistance markers and population structure [9].

Within the WHO Western Pacific Region, malaria cases have declined from 2,678,000 in 2000 to 1,747,000 in 2023 [1]. Malaysia has contributed significantly to this reduction and, as of 2018, has reported zero indigenous cases of non-zoonotic malaria [10]. Excluding cases of zoonotic malaria caused by *P. knowlesi*, monoclonal *P. vivax* infections were the predominant malaria cases reported in Malaysia before elimination [11]. However, as expected *P. falciparum* was associated with a higher average case fatality rate, 1.41% compared to just 0.04% for *P. vivax* [11]. Imported malaria made up 17.7% of all cases in Malaysia from 2013 to 2017 [11]. This is partly due to Malaysia’s reliance on migrant workers, especially from countries in the wider Asia region [12]. The majority of the migrant worker population originates from neighbouring high-risk countries, such as Indonesia and Thailand, where multiple drug-resistant malaria strains, including artemisinin-resistant *P. falciparum* from the Greater Mekong Subregion, have emerged [13, 14]. These populations represent a potential reservoir for the reintroduction of malaria.

Although non-zoonotic malaria has been eliminated in Malaysia, the zoonotic species *P. knowlesi* continues to pose a public health challenge, particularly in East Malaysia, where 2,879 related cases were reported in 2023 [1]. The transmission of *P. knowlesi* is positively associated with forest cover and historical forest loss, implicating deforestation driven by agriculture and forestry as key drivers of transmission from the macaque monkey host to humans [15]. The presence of well-established competent vectors underscores the real risk of re-introducing *P. falciparum* malaria transmission in the country, and the fragility of sustaining malaria elimination. The continued decline in malaria cases can lead to reduced public awareness, diminished health system preparedness, and potentially waning population immunity [16, 17]. Given the severe health consequences posed by *P. falciparum*, particularly among immunologically naïve populations [18], preventing its re-establishment, especially of drug-resistant strains, is an urgent public health priority. To inform such efforts, it is crucial to understand the historical *P. falciparum* population structure and dynamics. This requires the integration of WGS, population genomic analyses [7, 19], and advanced AI-based profiling approaches [20].

We performed ONT WGS and population genomics analysis on *P. falciparum* isolates from 21 patients with travel histories, sampled at two tertiary hospitals in Sarawak, Malaysia, between 2008 and 2010. Our objective was to assess the utility of genomic data in reconstructing the historical epidemiology of *P. falciparum* cases in the region. This work contributes to the development of a genomic reference database and analytical framework to support future whole-genome or amplicon-based surveillance efforts for malaria control in Malaysia and the wider region.

## Methods

### Sample collection, DNA extraction, and sequencing

A total of twenty-one whole blood samples (WBS) were collected from patients diagnosed with *P. falciparum* malaria and treated at Hospital Sarikei and Hospital Sibu in Sarawak, Malaysia, between 2008 and 2010 [21]. Nationwide, 21,330 malaria cases were diagnosed during this period. Informed consent, including that to use residual samples for related studies, was obtained from all participants, and individual travel histories were recorded.

Initial case identification and species determination were performed by microscopy and subsequently confirmed by quantitative PCR (qPCR). Genomic DNA was extracted from all twenty-one samples using the Monarch^®^ Genomic DNA Purification Kit (NEB, UK), which demonstrated broadly superior performance compared to the DNeasy^®^ Blood and Tissue Kit (Qiagen, UK) in an initial evaluation of twelve samples (Fig. S1, Table S1). The resulting DNA underwent SWGA using EquiPhi29™ DNA polymerase (Thermo Fisher Scientific) and primer sets previously described [22]. DNA concentration was measured using Qubit™ fluorometry, and sequencing libraries were prepared with the Native Barcoding Kit 24 V14 (SQK-NBD114.24, ONT, UK), following the manufacturer’s protocol. Isolates were multiplexed and sequenced on R10.4.1 flow cells for 24 to 48 h via The Applied Genomics Centre at the London School of Hygiene & Tropical Medicine (genomics.lshtm.ac.uk).

### Bioinformatics and genotypic profiling

Raw signal sequence data (.*pod5* files) underwent basecalling and demultiplexing using *dorado* (v0.7.3) software (ONT, UK) with the superior accuracy model (dna_r10.4.1_e8.2_400bps_sup@v5.0.0). Raw reads were trimmed using the in-built function in *dorado*, and alignment to the *P. falciparum* 3D7 (PF3D7; PlasmoDB; [23]) reference genome was performed using *minimap2* software (v2.1.1-r341) [24]. Raw sequence reads were also quality-checked by *pycoQC* (v2.5.2) and *NanoStat* (v1.6.0) [25]. Assembly depth across the core *P. falciparum* genome [26], excluding subtelomeric regions, was quantified using *mosdepth* software (v0.3.3) [27]. Isolates with less than 50% genome coverage at 5-fold coverage depth were removed from further analysis.

Raw *fastq* files were analysed using *Malaria-Profiler* software (v.0.0.10) [6], leading to predictions of likely geographical region source (Oceania, Southeast Asia, or Central Africa) and genotypic drug resistance. Raw sequencing data are available from the ENA archive (see Table S2 for a list of accession numbers). Variant calling of ONT data was performed by *Clair3* (v1.0.10) software with model r1041_e82_400bps_sup_v500 to create a GVCF output [28]. The SNP data obtained using Clair3 in GVCF format were merged with a database of 9,518 *P. falciparum* isolates from Central Africa (518), East Africa (849), Horn of Africa (25), Oceania (349), South America (75), South Asia (404), Southeast Asia (5,182) and West Africa (2,116) that were previously sequenced using an Illumina platform, obtained from the Pf7 data release [29]. The merge was performed using GATK’s GenomicsDBImport [30]. In total, 1,178 isolates (22.69%) were sequenced and assembled between 2008 and 2010, with a further 7,847 sequenced after 2010. The resulting variant file was filtered through an in-house pipeline (LSHTMPathogenSeqLab/fastq2matrix). In brief, *GATK* variant filtration (*30*; best practices), biallelic SNP selection, and *bcftools* [31] consequence calling for gene annotation, and 1% minor allele frequency filtering were applied (Table S3). The filtered variant file was taken forward for population genomic analysis, which excluded SNPs with high genotype missingness (threshold of 40%).

### Population genomic analyses

The population structure of the isolates was analysed using principal component analyses based on a SNP distance matrix generated by PLINK (v1.90b6.21) software [32] (https://github.com/LSHTMPathogenSeqLab/malaria-hub). Multiplicity of infection (MOI) was estimated by *Moimix* (v0.0.2) [33], which calculates Wright’s inbreeding coefficient (F_WS_), with values > 0.95 indicating monoclonality. Identity-by-descent (IBD) was estimated from SNP data using *hmmIBD* (v2.1.0), and was restricted to samples classified as monoclonal (F_WS_ > 0.95) [34]. Sample clusters were inferred for isolates that shared a minimum of 90% identity. IBD was estimated between Malaysian (*n* = 6) and monoclonal samples from Pf7 (*n* = 7,134).

## Results

### Patient and isolate characteristics

Twenty-one WBS containing *P. falciparum* were included in this study, collected from patients at Sibu (*n* = 17) and Sarikei (*n* = 4) district hospitals in Sarawak, Malaysia, in 2008 (*n* = 11), 2009 (*n* = 7) and 2010 (*n* = 3). The patients ranged in age from 19 to 52 years and were predominantly male (19/21; 90.5%), with the majority belonging to the Dayak indigenous communities (13/21; 61.9%) (Table 1). Parasitaemia levels varied from 0.01% to 8.8%, with a mean of 1.74% and a median of 1.0%. Travel history data indicated that eight patients had recently returned from Africa (Democratic Republic of the Congo (*n* = 2), Ghana (*n* = 1), South Africa (*n* = 1), other (*n* = 4)) and five from Oceania (Papua New Guinea (*n* = 4), Western New Guinea (*n* = 1)). Six patients reported no recent travel, and two others provided no travel history.

**Table 1:** Patient characteristics linked to their respective *Plasmodium* isolates (*n* = 21)*

Sample ID	Age (years)	Community Group	Hospital	Collection year	Parasitaemia (%)	Travel history
Pf_01	32	Chinese	Sibu	2008	1.66	Not reported
Pf_02	38	Dayak indigenous	Sibu	2008	0.97	PNG (Oceania)
Pf_03	33	Filipino	Sibu	2008	0.62	Not reported
Pf_04	44	Chinese	Sibu	2008	8.80	WNG (Oceania)
Pf_05	49	Dayak indigenous	Sibu	2008	1.60	Africa
Pf_06	37	Dayak indigenous	Sibu	2008	2.56	No travel
Pf_07	34	Dayak indigenous	Sibu	2008	0.12	Africa
Pf_08	24	Dayak indigenous	Sarikei	2008	2.40	No travel
Pf_09	19	Dayak indigenous	Sarikei	2009	0.14	No travel
Pf_10	43	Dayak indigenous	Sibu	2008	0.01	Ghana (Africa)
Pf_11	29	Chinese	Sibu	2008	1.05	No travel
Pf_12	43	Dayak indigenous	Sibu	2008	3.58	PNG (Oceania)
Pf_13	22	Malay	Sarikei	2009	4.19	No travel
Pf_14	28	Malay	Sibu	2009	0.08	DRC (Africa)
Pf_15	19	Dayak indigenous	Sibu	2009	0.02	No travel
Pf_16	25	Chinese	Sibu	2009	0.38	DRC (Africa)
Pf_17	47	Chinese	Sibu	2009	1.30	PNG (Oceania)
Pf_18	40	Dayak indigenous	Sibu	2009	2.72	PNG (Oceania)
Pf_19	50	Dayak indigenous	Sibu	2010	0.01	Africa
Pf_20	52	Dayak indigenous	Sarikei	2010	0.11	South Africa (Africa)
Pf_21	31	Dayak indigenous	Sibu	2010	2.43	Africa

*PNG* Papua New Guinea, *DRC* Democratic Republic of the Congo, *WNG* Western New Guinea

* all confirmed with *P. falciparum * infections using PCR and microscopy

### Sequencing and genotypic profiles

The twenty-one *P. falciparum* DNA samples were sequenced using the ONT PromethION platform. The median sequencing depth across genome positions was 9.12-fold. The median percentage of the core genome covered at a minimum depth of 5-fold was 72.8% (range: 0.3% to 98.3%) (Table S4).

Of the 21 samples, 15 met the predefined threshold of ≥ 50% genome coverage at 5-fold depth with ≤ 40% missing genotypes and were included in downstream population genomic analyses.

Drug resistance profiling was performed using *Malaria-Profiler* software. Twelve of the 15 isolates harboured one or more drug resistance-associated genotypes (Table 2, Table S5). Chloroquine resistance was the most frequently observed, with ten isolates carrying at least one mutation in the *pfcrt* gene, and five of these also possessed mutations in *pfmdr1*. The most common *pfcrt* mutation was A220S (8/10 isolates), while N86Y was the dominant *pfmdr1* variant (4/5). Resistance-associated mutations in *pfpppk-dhps* (sulfadoxine) and *pfdhfr-ts* (pyrimethamine) were identified in seven isolates each. The most prevalent markers were *pfpppk-dhps* A437G and *pfdhfr-ts* S108N. Five isolates (Pf_06, Pf_07, Pf_11, Pf_12, and Pf_17) carried genotypes associated with resistance to both chloroquine and sulfadoxine-pyrimethamine (SP). In contrast, three isolates (Pf_09, Pf_15, Pf_20) displayed no known resistance mutations and were classified as drug-sensitive. No artemisinin resistance-associated genotypes were detected.

**Table 2:** Genotypic drug resistance profiles and geographical classification produced by *Malaria-Profiler* and multiplicity of infection of the Malaysian isolates. Isolates were considered monoclonal if their F_ws_ score was > 0.95

Sample ID	Drug resistance	Gene (mutations)	Geographical classification (Highest predictive probabilities)	F_ws_ score*
Pf_02	Chloroquine	*pfcrt* (A220S, C72S, K76T) *pfmdr1* (N1042D, Y184F)	SEA (0.50) and Oceania (0.28)	0.93
	Pyrimethamine	*pfdhfr-ts* (C59R, S108N)		
Pf_03	Chloroquine	*pfcrt* (A220S, C72S, K76T)	Oceania (1.00) and SEA (0.001)	0.92
Pf_05	Chloroquine	*pfcrt* (A220S)	SEA (0.61) and South Asia (0.36)	0.96
Pf_06	Chloroquine	*pfcrt* (A220S, R371I, Q271E, I356T, K76T, M74I)	Central Africa (0.91) and West Africa (0.09)	0.92
		*pfmdr1* (N86Y)		
	Pyrimethamine	*pfdhfr-ts* (S108N)		
	Sulfadoxine	*pfpppk-dhps* (A437G, I431V, S436A)		
Pf_07	Chloroquine	*pfcrt* (R371I, Q271E, I356T, K76T, M74I)	Central Africa (0.91) and West Africa (0.09)	0.97
		*pfmdr1* (N86Y, Y184F)		
	Pyrimethamine	*pfdhfr-ts* (N51I, C59R, S108N)		
	Sulfadoxine	*pfpppk-dhps* (A437G, I431V, S436A)		
Pf_08	Chloroquine	*pfcrt* (A220S, C72S, K76T)	SEA (0.55) and South Asia (0.36)	0.96
Pf_09	Sensitive	N/A	Oceania (0.93) and SEA (0.06)	0.90
Pf_11	Chloroquine	*pfcrt* (C72S, K76T)	Oceania (0.98) and SEA (0.01)	0.94
		*pfmdr1* (N86Y)		
	Pyrimethamine	*pfdhfr-ts* (I164L)		
	Sulfadoxine	*pfpppk-dhps* (A437Gn, A581G)		
Pf_12	Chloroquine	*pfcrt* (A220S, C72S, K76T)	Oceania (1.0) and Horn of Africa (0.0006)	0.96
	Pyrimethamine	*pfdhfr-ts* (S108N)		
	Sulfadoxine	*pfpppk-dhps* (A437G)		
Pf_13	Chloroquine	*pfcrt* (A220S)	SEA (0.46) and South Asia (0.39)	0.96
	Pyrimethamine	*pfdhfr-ts* (S108N)		
Pf_15	Sensitive	N/A	SEA (0.80) and South Asia (0.09)	0.97
Pf_17	Chloroquine	*pfcrt* (A220S, C72S, K76T)	Oceania (0.98) and SEA (0.02)	0.79
		*pfmdr1* (N86Y)		
	Pyrimethamine	*pfdhfr-ts* (C59R, S108N)		
	Sulfadoxine	*pfpppk-dhps* (A437G)		
Pf_18	Sulfadoxine	*pfpppk-dhps* (A437G)	Oceania (0.99) and SEA (0.004)	0.81
Pf_20	Sensitive	N/A	Central Africa (0.64) and West Africa (0.36)	0.70
Pf_21	Sulfadoxine	*pfpppk-dhps* (A437G)	Central Africa (0.92) and West Africa (0.06)	0.92

*SEA* Southeast Asia, *N/A* not applicable

* the score of 1 indictates monoclonality and 0, polyclonal infection

### Genotypic source, travel histories, and population structure

Based on the AI-driven geographical prediction model implemented in *Malaria-Profiler* software, the fifteen *P. falciparum* isolates were classified as originating from Central Africa (*n* = 4), Southeast Asia (*n* = 5), and Oceania (*n* = 6) (Table 2). When compared with recorded patient travel histories, the predicted geographical origins were concordant for all but two isolates. Isolate Pf_05 was predicted to originate from Southeast Asia, despite the patient having a travel history to Africa. Conversely, isolate Pf_06, from a patient with no reported travel history, was classified as originating from Central Africa.

**Fig. 1:**
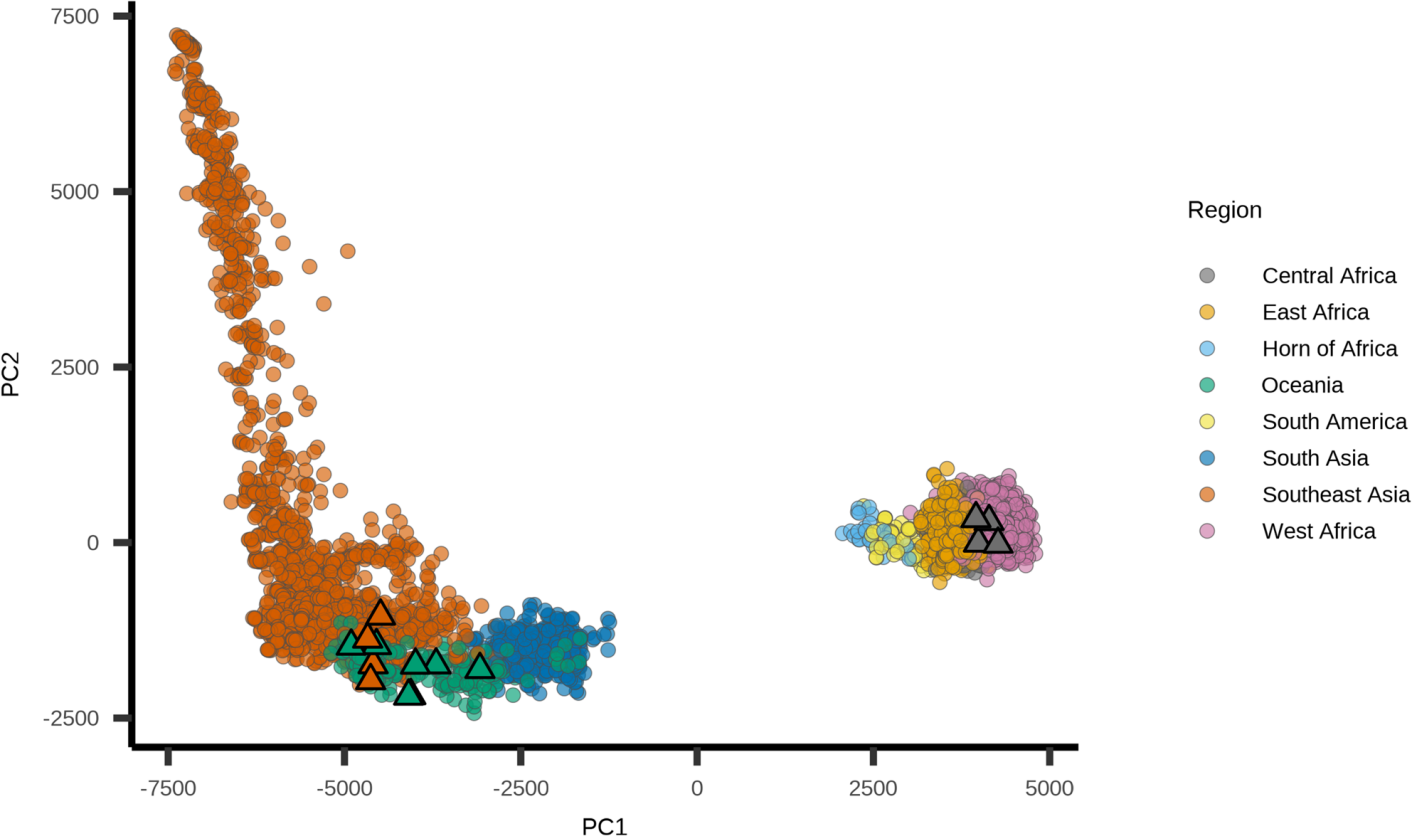
Principal component analysis (PCA) combined with multidimensional scaling (MDS) demonstrated that Malaysian isolates cluster with a curated *P. falciparum* reference database. The global dataset comprised 9,518 *P. falciparum* isolates from 32 countries across eight geographical regions. The PCA plot is shown by geographical region, with Malaysian isolates denoted by a Δ and coloured according to their predicted regions. These isolates clustered within the regions consistent with patients’ reported travel histories

To further contextualise the origin of these isolates, we positioned them within a global *P. falciparum* reference dataset (*n* = 9,518). A total of 4,685,356 SNPs were identified from the initial dataset, including 4,391,371 biallelic SNPs across 14 nuclear chromosomes. Following filtering, 1,785,208 biallelic SNPs remained for further analysis. Principal component analysis (PCA) using multidimensional scaling revealed the expected clustering of isolates by geographic region, with some overlap between isolates from Oceania and Southeast Asia. The Malaysian isolates clustered by their *Malaria-Profiler*-predicted origins (Fig. 1), reinforcing the validity of the AI-based classifications. Notably, the overlapping positions of some Southeast Asian and Oceanian isolates may reflect the geographic proximity of Sarawak to neighbouring regions, including the Indonesian state of Kalimantan, and the potential for cross-border malaria transmission or shared parasite ancestries.

### Malaysian isolates presented with potential multiclonal infections

To investigate the likely transmission intensity at the sites of infection, the within-host diversity metric F_WS_​ was estimated. Values greater than 0.95 were considered indicative of monoclonal infections. The estimated F_WS_​ scores ranged from 0.70 to 0.97, with a mean of 0.91, suggesting that many infections likely originated from areas with moderate to high transmission. Evidence of polyclonality was observed in nine of the fifteen Malaysian isolates, each exhibiting F_WS_​ values ≤0.95 (Fig. S2, Table 2).

Stratified by predicted geographical origin, most monoclonal isolates were assigned to Southeast Asia (4 of 6), with the remainder from Oceania and Central Africa. The predominance of monoclonality among Southeast Asian isolates aligns with known low transmission intensities and declining *P. falciparum* prevalence in the region, particularly in countries such as Thailand and Vietnam, which were actively pursuing malaria elimination during the study period [35]. In contrast, the higher prevalence of polyclonal infections among isolates associated with Central Africa and Oceania is consistent with historically and currently high malaria transmission intensities and large *P. falciparum* parasite reservoirs in those regions [1].

### Identity-by-descent analysis

**Fig. 2:**
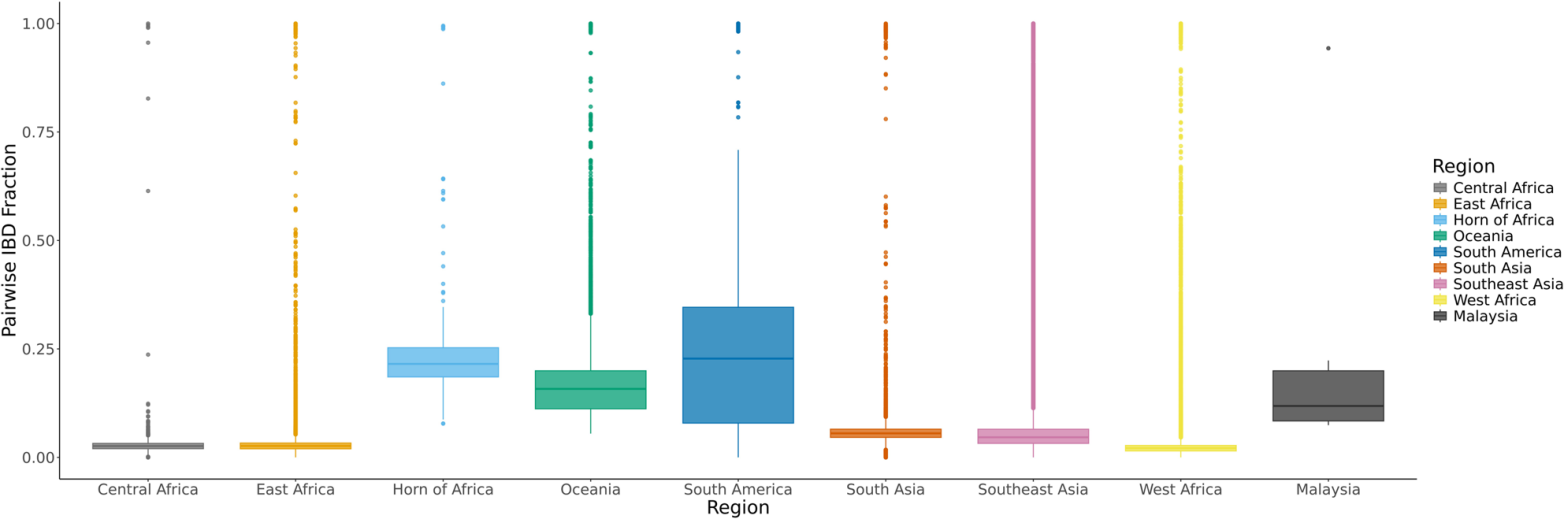
Pairwise identity-by-descent (IBD) sharing between Malaysia and global regions. Distribution of pairwise IBD fractions calculated between individuals from Malaysia (*n* = 6) and individuals from eight global regions (*n* = 7,134). The x-axis denotes the comparison region, and the y-axis shows the pairwise IBD fraction. Colours indicate geographical regions: Central Africa (*n* = 212), East Africa (*n* = 619), Horn of Africa (*n* = 16), Oceania (*n* = 271), South Asia (*n* = 310), Southeast Asia (*n* = 4,030), West Africa (*n* = 1,615), and South America (*n* = 61). All samples were filtered to include only monoclonal infections (FWS > 0.95). Higher IBD fractions indicate more recent shared ancestry between Malaysian samples and individuals from the corresponding region

Segments of IBD were subsequently used to assess genetic relatedness between monoclonal isolates from Malaysia (*n* = 6) and those from eight geographical regions (*n* = 7,134) (Fig. 2). Overall, comparisons between samples from Malaysia revealed high pairwise IBD fractions (median: 0.11, range: 0.07–0.94). Samples from Malaysia showed low IBD with isolates from the same timeframe in the database (median pairwise IBD < 0.1 across all eight regions; Fig. 2). This was higher than other regions including West Africa (median: 0.02, range: 0–1), Central Africa (median: 0.03, range: 0–1), East Africa (median: 0.03, range: 0–1), Southeast Asia (median: 0.05, range: 0–1) and South Asia (median: 0.06, range: 0–0.99), but similar to Oceania (median: 0.16, range: 0.05–1), consistent with six samples predicted to be from this region (Fig. 2). Two isolates, Pf_08 and Pf_13 had the highest IBD proportions (90.3%), suggesting possible shared common ancestry. The two patients from whom these isolates were obtained reported no travel history, were of Malaysian ethnicity (Bidayuh and Malay), and genomic analyses classified the isolates as originating from Southeast Asia. This result supports the likelihood that the infections were acquired locally, suggesting ongoing transmission within the region. Although both isolates were genotypically resistant to chloroquine, differences in their resistance genotypes point to potentially distinct drug selective pressures.

## Discussion

Malaysia is no longer considered a malaria-endemic country, with zero non-zoonotic indigenous cases reported for 6 consecutive years (2018–2023) [1]. Population mobility poses a significant challenge to preventing malaria re-establishment and threatens Malaysia’s recent successes, particularly in East Malaysia, through disease reintroduction via importation-mediated outbreaks derived from the global demand for skilled workers in agricultural, forestry and industrial sectors [36]. In this study, we leveraged long-read nanopore sequencing to perform retrospective genomic surveillance from archived WBS. Through this analysis, it was possible to elucidate the drug resistance prevalence, demographics of potential imported malaria cases, and the approximate regional transmission intensities within that time frame; all of which are vital towards securing malaria elimination, especially in preventing re-introduction. Such knowledge has proven important towards malaria control programmes, including tailoring drug strategies [13].

Geographical classification was derived from the genomic information from the Malaysian archived isolates and identified potential case importations. Frequent travels for employment have often been cited as a major mode for malaria influx from high malaria transmission zones [37, 38]. Most cases were found to cluster closely with their geographical counterpart, particularly for those of possible Central African origins. Other studies conducted in Malaysia further reinforce these findings [10, 12, 39], with Papua New Guinea, Indonesia, Nigeria and Pakistan being sources of imported cases [10]. Two cases were contradictory to the patients’ travel histories but could be explained by exposure to local strains or inaccurate self-reported travel. The high accuracy of malaria genomic-based geographical profiling, which can distinguish local from imported infections, provides a valuable complement to traditional epidemiological approaches that rely on self-reported travel histories - data that are often incomplete or unreliable, yet remain the primary source guiding immediate clinical management and public health responses [38]. As sequencing data continues to grow, genomic-based predictions can be refined to the country level, helping to resolve ambiguities between Southeast Asian and Oceanian isolates. Crucially, the detection of imported drug-resistant parasites, including *pfkelch13* mutations that vary regionally, will provide critical insights for targeted infection control efforts. Similarly, our estimates of polyclonality, or multiplicity of infection, reflected the transmission intensity in the inferred source countries at the time of collection [40], and further supported regional geographical classifications, such as the higher transmission levels observed in Africa.

From the drug resistance profiles, the majority of the Malaysian isolates in this study were found to carry resistance genotypes to chloroquine, sulfadoxine, and pyrimethamine, including presently common mutant alleles in *pfmdr1* and *pfcrt* genes. The drug resistance profiles from this study follow the documented malaria epidemiology and national drug policies of the regions [41]. Interestingly, no artemisinin resistance genotypes were found in the isolates despite their strong presence throughout Southeast Asia during the collection period [42]. However, the sample size is small, and there are biological and fitness cost barriers hindering the spread of artemisinin resistance [43], especially through importation.

Importation events can also be corroborated through ancestry clustering using IBD analysis, which revealed no genetic relatedness among the isolates except for Pf_08 and Pf_13, suggesting that these two may belong to a common transmission cluster. Although the sampling was not exhaustive, the overall findings suggest that most of the sequenced isolates likely represent independent transmission events over the study period. Similar patterns have been reported in other studies, where *P. falciparum* population genetic heterogeneity is more often associated with importation, whereas genetic relatedness typically reflects ongoing local transmission [40]. The lack of clustering between the isolates and publicly available sequences is likely due to the absence of representative samples from the actual source regions of the infections. This underscores the need for broader genomic surveillance in malaria-endemic regions, particularly among at-risk groups.

Moreover, whilst we have successfully demonstrated the utility of ONT sequencing to inform the epidemiology and surveillance of *P. falciparum* during the pre-elimination stage in Malaysia, future improvements in ONT sequencing yield and genomic coverage will be essential to enhance such approaches in the future. This may explain the minor underlying differences in geographical classification and travel history reported. Additionally, the nanopore-sequenced *P. falciparum* genomes were compared to the curated MalariaGEN Pf7 dataset, composed of Illumina-sequenced *P. falciparum* isolates. Although care was taken to select a relevant dataset, the use of different sequencing platforms and convenience sampling could affect geographical classification and IBD analysis. Overcoming these limitations, increasing genome coverage, as well as spatiotemporal and geographical resolution of training datasets, will enhance ONT-based surveillance efforts in the future.

Overall, this study demonstrates the utility of malaria genomics, specifically through long-read sequencing technology, in distinguishing imported *P. falciparum* cases from archived WBS. It also highlights the value of population genomic analyses in complementing traditional malaria epidemiological investigations. Although retrospective and small-scale in nature, the ability to differentiate imported cases reinforces Malaysia’s malaria elimination trajectory and underscores the epidemiological relevance of such distinctions. These findings further emphasise the urgent need to integrate genomic data into elimination strategies, particularly to support surveillance among high-risk mobile populations, where patient travel histories are often incomplete or unreliable. With continued advancements in sequencing throughput, genome coverage, and reductions in cost, genomic pipelines are becoming increasingly feasible for implementation, even in resource-limited settings. Importantly, the successful use of archived WBS illustrates the robustness of malaria genomics for retrospective analyses. The end-to-end workflow from genetic sequencing to population genomic analysis has demonstrated the operational practicality of third-generation sequencing technologies combined with open-source bioinformatics tools. As malaria elimination efforts progress, preventing the reintroduction and onward transmission of imported parasites will require that genomic surveillance becomes an integral component of both national and global malaria control strategies. 

## Supplementary Information

Additional file 1: Figure S1. DNA concentration (A) and yield per sample (B) obtained from DNeasy® Blood and Tissue Kit (Qiagen, UK) and Monarch® Genomic DNA Purification Kit (NEB, UK). 12 frozen WBS were extracted with both kits and compared against each other with the Monarch® Genomic DNA Purification Kit (NEB, UK) showing better extraction yields both in DNA concentration and total DNA yield. The remaining nine whole blood samples (WBS) from this study were extracted with this kit. Figure S2. Estimated multiplicity of infection (MOI) using the within-host diversity metric FWS*. × denotes Malaysian isolates with their designated source region; * the score of 1 dictates monoclonality and 0, polyclonal infection. Table S1. DNA concentration and yield obtained from both extraction kits, DNeasy® Blood and Tissue Kit (Qiagen, UK) and Monarch® Genomic DNA Purification Kit (New England Biolabs, UK). Table S2. ENA Accession numbers for the project PRJEB90161. Table S3. Number of nuclear chromosome variants remaining following each filtration step. Table S4. Sequencing yields, assembly metrics and genomic coverage. Table S5. Regional frequencies of drug resistance genotypes and Malaysian isolates with respective genotypes

Additional file 2: Table S6. List of accession numbers and details of curated dataset used.

## Data Availability

Raw sequencing data is available from the ENA archive (PRJEB90161; see Table S2 for a list of accession numbers). Details of the curated dataset are contained in Table S6.
