# Supplementary material for: Whole genome sequencing to inform the epidemiology of Plasmodium falciparum malaria in the elimination setting of Malaysia
Source: BMC Genomics. 2026 Feb 20;27:313. doi: 10.1186/s12864-026-12653-7 (PMC13032384; doi:10.1186/s12864-026-12653-7)
Supplement: Supplementary file 1 — Additional file 1: Figure S1. DNA concentration (A) and yield per sample (B) obtained from DNeasy® Blood and Tissue Kit (Qiagen, UK) and Monarch® Genomic DNA Purification Kit (NEB, UK). 12 frozen WBS were extracted with both kits and compared against each other with the Monarch® Genomic DNA Purification Kit (NEB, UK) showing better extraction yields both in DNA concentration and total DNA yield. The remaining nine whole blood samples (WBS) from this study were extracted with this kit. Figure S2. Estimated multiplicity of infection (MOI) using the within-host diversity metric FWS*. × denotes Malaysian isolates with their designated source region; * the score of 1 dictates monoclonality and 0, polyclonal infection. Table S1. DNA concentration and yield obtained from both extraction kits, DNeasy® Blood and Tissue Kit (Qiagen, UK) and Monarch® Genomic DNA Purification Kit (New England Biolabs, UK). Table S2. ENA Accession numbers for the project PRJEB90161. Table S3. Number of nuclear chromosome variants remaining following each filtration step. Table S4. Sequencing yields, assembly metrics and genomic coverage. Table S5. Regional frequencies of drug resistance genotypes and Malaysian isolates with respective genotypes [file 12864_2026_12653_MOESM1_ESM.docx]

**Supplementary Information**

**Figure S1**. DNA concentration (**A**) and yield per sample (**B**) obtained from DNeasy® Blood and Tissue Kit (Qiagen, UK) and Monarch® Genomic DNA Purification Kit (NEB, UK). 12 frozen WBS were extracted with both kits and compared against each other with the Monarch® Genomic DNA Purification Kit (NEB, UK) showing better extraction yields both in DNA concentration and total DNA yield. The remaining nine whole blood samples (WBS) from this study were extracted with this kit.

**
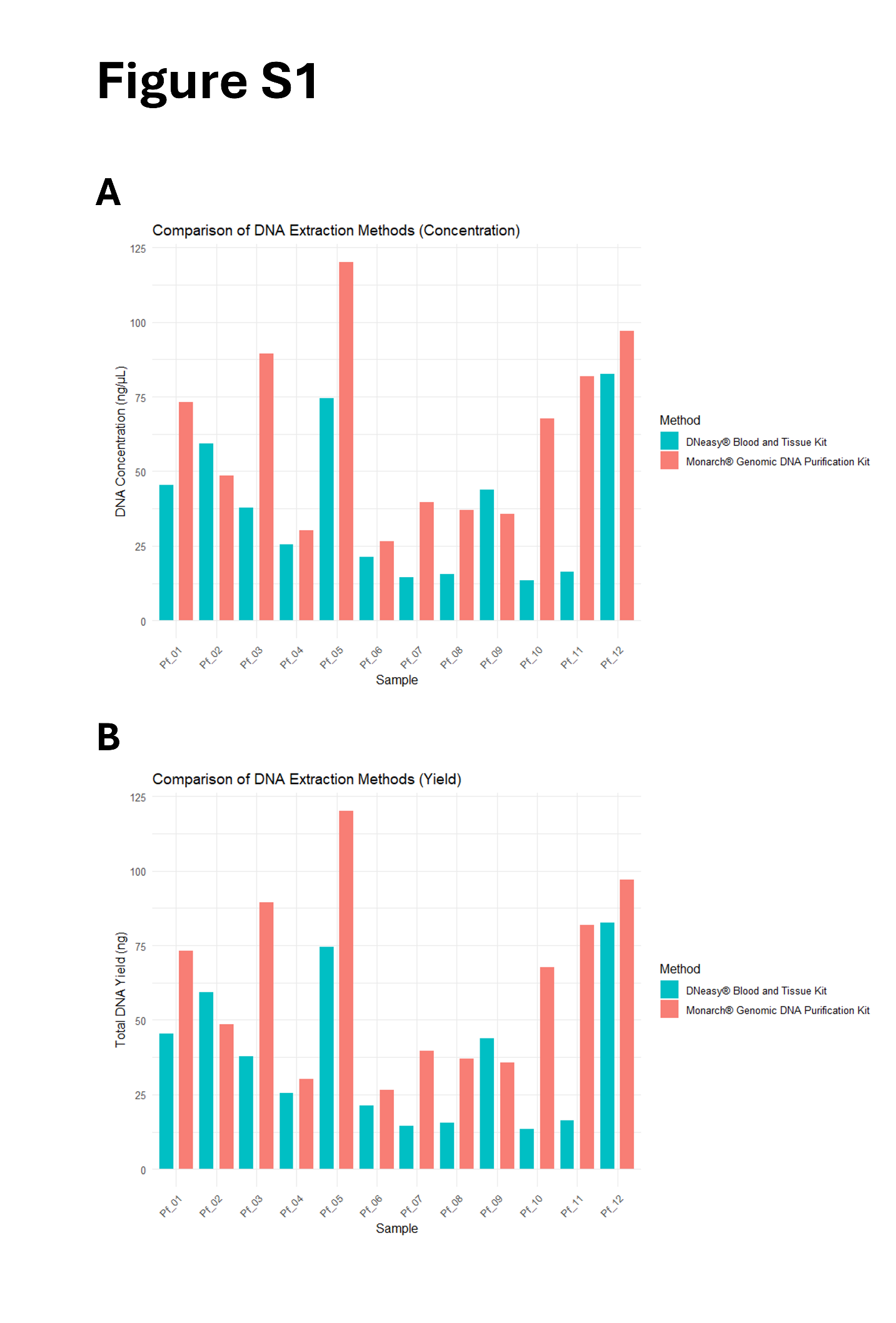
**

**Figure S2**. Estimated multiplicity of infection (MOI) using the within-host diversity metric F_WS_*. × denotes Malaysian isolates with their designated source region; * A score of 1 denotes monoclonality, whereas a score of 0 denotes a polyclonal infection.

**
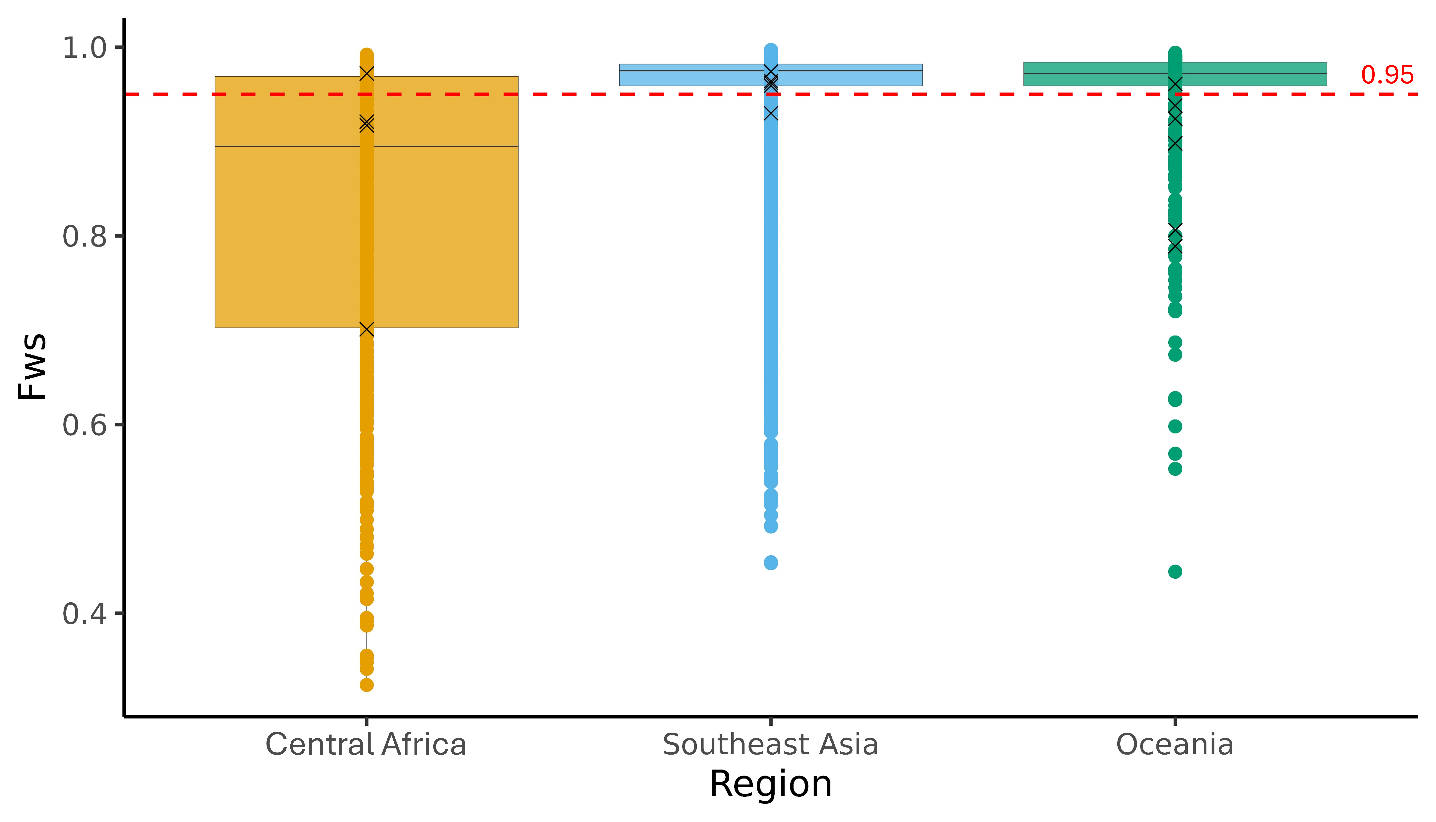
**

**Table S1**. DNA concentration and yield obtained from both extraction kits, DNeasy® Blood and Tissue Kit (Qiagen, UK) and Monarch® Genomic DNA Purification Kit (New England Biolabs, UK)

| Sample  Number | DNA concentration (ng/µL) | | Total DNA yield (ng) | |
| --- | --- | --- | --- | --- |
|  | DNeasy® | Monarch® | DNeasy® | Monarch® |
| Pf_01 | 45.5 | 73.1 | 1365 | 2558.5 |
| Pf_02 | 59.4 | 48.5 | 1782 | 1697.5 |
| Pf_03 | 37.8 | 89.4 | 1134 | 3129 |
| Pf_04 | 25.5 | 30.1 | 765 | 1053.5 |
| Pf_05 | 74.6 | 120 | 2238 | 4200 |
| Pf_06 | 21.3 | 26.6 | 639 | 931 |
| Pf_07 | 14.4 | 39.5 | 432 | 1382.5 |
| Pf_08 | 15.6 | 37.0 | 468 | 1295 |
| Pf_09 | 43.8 | 35.8 | 1314 | 1253 |
| Pf_10 | 13.3 | 67.8 | 399 | 2373 |
| Pf_11 | 16.4 | 81.8 | 492 | 2863 |
| Pf_12 | 82.6 | 97.1 | 2478 | 3398.5 |

**Table S2.** ENA Accession numbers for the project PRJEB90161

| Sample ID | Accession number |
| --- | --- |
| Pf_01 | ERS24831098 |
| Pf_02 | ERS24831089 |
| Pf_03 | ERS24831099 |
| Pf_04 | ERS24831093 |
| Pf_05 | ERS24831081 |
| Pf_06 | ERS24831097 |
| Pf_07 | ERS24831090 |
| Pf_08 | ERS24831100 |
| Pf_09 | ERS24831080 |
| Pf_10 | ERS24831091 |
| Pf_11 | ERS24831095 |
| Pf_12 | ERS24831083 |
| Pf_13 | ERS24831096 |
| Pf_14 | ERS24831086 |
| Pf_15 | ERS24831085 |
| Pf_16 | ERS24831092 |
| Pf_17 | ERS24831088 |
| Pf_18 | ERS24831094 |
| Pf_19 | ERS24831084 |
| Pf_20 | ERS24831082 |
| Pf_21 | ERS24831087 |

**Table S3**. Number of nuclear chromosome variants remaining following each filtration step

| File | Total variant site count | Biallelic SNP sites | Multiallelic SNP sites | Total number of SNPs alleles | Total number of indels |
| --- | --- | --- | --- | --- | --- |
| Unfiltered VCF | 5,937,810 | 4,391,371 | 293,985 | 4,685,356 | 2,373,176 |
| Filtered VCF | 2,207,190 | 2,033,437 | 173,753 | 2,207,190 | 0 |
| Biallelic SNP-only VCF | 1,785,208 | 1,785,208 | 0 | 1,785,208 | 0 |

Indels: Insertions and deletions alleles; Total variant site counts indicate the number of VCF variant sites, also encompassing biallelic and multiallelic sites while SNPs and indels refer to alternate allele counts, which may both occur at the same variant site.

**Table S4**. Sequencing yields, assembly metrics and genomic coverage*

| Sample  ID | Total raw sequencing reads obtained | Total number of DNA bases | Raw sequencing read N50 | Mean raw sequencing read quality | Percentage core genome coverage at 5-fold coverage depth | Percentage core genome coverage at 10-fold coverage depth | Median genome coverage depth (fold) |
| --- | --- | --- | --- | --- | --- | --- | --- |
| Pf_01** | 64,341 | 167,834,280 | 6,063 | 18.3 | 38.5% | 12.6% | 3.9 |
| Pf_02 | 264,424 | 767,199,806 | 7,097 | 18.0 | 98.3% | 91.6% | 24.5 |
| Pf_03 | 196,913 | 484,963,122 | 4,543 | 19.2 | 72.8% | 45.9% | 9.1 |
| Pf_04** | 109,552 | 292,911,137 | 4,826 | 19.3 | 44.8% | 19.1% | 4.4 |
| Pf_05 | 142,976 | 334,752,989 | 4,246 | 18.8 | 77.8% | 46.7% | 9.4 |
| Pf_06 | 175,212 | 398,313,956 | 4,631 | 18.5 | 81.1% | 52.1% | 10.4 |
| Pf_07 | 217,810 | 568,043,751 | 6,076 | 18.1 | 91.5% | 71.8% | 14.9 |
| Pf_08 | 137,009 | 450,480,774 | 5,648 | 19.8 | 87.3% | 62.3% | 12.4 |
| Pf_09 | 149,513 | 354,980,611 | 3,638 | 19.3 | 65.5% | 36.3% | 7.4 |
| Pf_10** | 301,956 | 866,424,840 | 4,415 | 19.6 | 4.0% | 0.4% | 0.6 |
| Pf_11 | 213,653 | 567,015,009 | 4,065 | 19.6 | 87.7% | 66.0% | 13.7 |
| Pf_12 | 438,993 | 1,091,264,513 | 4,680 | 18.3 | 91.4% | 77.7% | 19.6 |
| Pf_13 | 181,284 | 494,596,212 | 4,466 | 19.6 | 91.9% | 73.6% | 15.3 |
| Pf_14** | 143,103 | 344,136,233 | 4,107 | 19.4 | 40.9% | 19.3% | 4.0 |
| Pf_15 | 228,425 | 534,287,261 | 3,512 | 19.6 | 61.7% | 36.7% | 7.0 |
| Pf_16** | 238,381 | 605,435,234 | 4,141 | 20.3 | 0.3% | 0.0% | 0.0 |
| Pf_17 | 194,630 | 551,499,147 | 4,655 | 19.7 | 84.3% | 61.4% | 12.6 |
| Pf_18 | 180,592 | 495,552,313 | 4,515 | 19.9 | 83.3% | 59.7% | 12.1 |
| Pf_19*** | 202,524 | 559,597,149 | 4,571 | 19.7 | 61.4% | 35.1% | 6.8 |
| Pf_20 | 230,930 | 487,307,332 | 3,189 | 19.5 | 70.4% | 45.5% | 9.0 |
| Pf_21 | 91,550 | 281,224,039 | 5,886 | 19.5 | 66.6% | 34.1% | 7.3 |

* Calculated after aligning the reads to the PF3D7 reference genome and removing non-core genomic regions; ** removed from population genomics analysis as <50% of the genome had 5-fold coverage; *** removed because of missing genotypes (≥40% missing SNPs from the biallelic SNP-only VCF)

**Table S5.** Regional frequencies of drug resistance genotypes and Malaysian isolates found with the respective genotypes.

| Drug | Gene | Mutation | Region | Regional % | Sample ID (region) |
| --- | --- | --- | --- | --- | --- |
| Chloroquine | *pfcrt* | A220S | SEA | 95.71% (4486/4687) | Pf_02 (SEA), Pf_05 (SEA), Pf_08 (SEA), Pf_13 (SEA) |
|  |  |  | OCN | 93.87% (306/326) | Pf_03 (OCN), Pf_12 (OCN), Pf_17 (OCN) |
|  |  |  | CA | 53.19% (200/376) | Pf_06 (CA) |
|  |  | C72S | OCN | 94.12% (320/340) | Pf_03 (OCN), Pf_11 (OCN), Pf_12 (OCN), Pf_17 (OCN) |
|  |  |  | SEA | 0.00% (0/5167) | Pf_02 (SEA), Pf_08 (SEA) |
|  |  | K76T | SEA | 96.08% (4953/5155) | Pf_02 (SEA), Pf_08 (SEA) |
|  |  |  | OCN | 94.13% (321/341) | Pf_03 (OCN), Pf_11 (OCN), Pf_12 (OCN), Pf_17 (OCN) |
|  |  |  | CA | 57.00% (293/514) | Pf_06 (CA), Pf_07 (CA) |
|  |  | R371I | CA | 56.56% (289/511) | Pf_06 (CA), Pf_07 (CA) |
|  |  | Q271E | CA | 56.00% (280/500) | Pf_06 (CA), Pf_07 (CA) |
|  |  | I356T | CA | 22.55% (115/510) | Pf_06 (CA), Pf_07 (CA) |
|  |  | M74I | CA | 0.00% (0/514) | Pf_06 (CA), Pf_07 (CA) |
|  | *pfmdr1* | N1042D | SEA | 1.06% (54/5102) | Pf_02 (SEA) |
|  |  | Y184F | SEA | 39.90% (2053/5145) | Pf_02 (SEA) |
|  |  |  | CA | 25.68% (132/514) | Pf_07 (CA) |
|  |  | N86Y | OCN | 36.36% (124/341) | Pf_11 (OCN), Pf_17 (OCN) |
|  |  |  | CA | 29.96% (154/514) | Pf_06 (CA), Pf_07 (CA) |
| Pyrimethamine | *pfdhfr-ts* | C59R | SEA | 99.13% (5114/5159) | Pf_02 (SEA) |
|  |  |  | OCN | 98.20% (328/334) | Pf_17 (OCN) |
|  |  |  | CA | 76.46% (393/514) | Pf_07 (CA) |
|  |  | S108N | SEA | 99.15% (5109/5153) | Pf_02 (SEA), Pf_13 (SEA) |
|  |  |  | OCN | 99.10% (332/335) | Pf_12 (OCN), Pf_17 (OCN) |
|  |  |  | CA | 99.22% (510/514) | Pf_06 (CA), Pf_07 (CA) |
|  |  | N51I | CA | 96.30% (494/513) | Pf_07 (CA) |
|  |  | I164L | OCN | 0.00% (0/339) | Pf_11 (OCN) |
| Sulfadoxine | *pfpppk-dhps* | A437G | OCN | 68.25% (230/337) | Pf_11 (OCN),  Pf_12 (OCN), Pf_17 (OCN), Pf_18 (OCN) |
|  |  |  | CA | 93.77% (482/514) | Pf_06 (CA), Pf_07 (CA), Pf_21 (CA) |
|  |  | I431V | CA | 0.78% (4/514) | Pf_06 (CA), Pf_07 (CA) |
|  |  | S436A | CA | 6.61% (34/514) | Pf_06 (CA), Pf_07 (CA) |
|  |  | A581G | OCN | 0.00% (0/341) | Pf_11 (OCN) |

SEA: Southeast Asia, OCN: Oceania, and CA: Central Africa; Note that the changes in total number of isolates per drug resistance gene mutations reflect genotype missingness.
